# Influence of Acute Beetroot Juice Intake on Agility Performance Immediately Post-Repeated Maximal Sprinting in Soccer Players

**DOI:** 10.3390/nu18060897

**Published:** 2026-03-12

**Authors:** Xueheng Yang, Takehira Nakao, Atsushi Saito

**Affiliations:** 1 Graduate School of Human-Environment Studies, Kyushu University, 744 Motooka, Nishi-ku, Fukuoka 819-0395, Japan; 2 Faculty of Human Science, Kyushu Sangyo University, 3-1 Matsukadai 2, Higashi-ku, Fukuoka 813-8503, Japan; takehira@ip.kyusan-u.ac.jp; 3 Faculty of Human-Environment Studies, Kyushu University, 744 Motooka, Nishi-ku, Fukuoka 819-0395, Japan; saito-a@ihs.kyushu-u.ac.jp

**Keywords:** agility, soccer players, beetroot juice, high-intensity exercise, nitrate

## Abstract

Background/Objectives: Acute beetroot juice (BJ) nitrate supplementation may enhance agility and change of direction performance by increasing nitric oxide bioavailability and improving cognitive and skeletal muscle functions, thereby potentially attenuating post-sprint performance decrements. Methods: We investigated whether a single dose of BJ improves agility immediately after repeated maximal sprinting compared with a placebo (PL) in soccer players. Twenty-one male soccer players (competitive players: *n* = 8; recreational players: *n* = 13) completed a double-blind randomised crossover trial. Participants ingested 70 mL of BJ or PL; afterwards, they performed three sets of 6 × 20-m all-out sprints. Agility outcomes included simple response time (SRT), choice response time (CRT), and change-of-direction speed (CODS). Non-parametric analyses (Wilcoxon signed-rank test with effect size r) were applied. Results: Compared with PL, BJ improved CRT (BJ: 2.376 ± 0.255; PL: 2.534 ± 0.322; *p* < 0.001; *r* = 0.74; *Z* = −5.881) and CODS (BJ: 13.046 ± 1.512; PL: 13.651 ± 1.811; *p* < 0.001; *r* = 0.47; *Z* = −4.314). SRT was unchanged overall (BJ: 1.671 ± 0.195; PL: 1.707 ± 0.261; *p* = 0.185; *r* = 0.05; *Z* = −1.327). Conclusions: Under practical field-based conditions, acute BJ intake enhanced post-sprint agility and change-of-direction performance, particularly CRT and CODS.

## 1. Introduction

During soccer matches, players must execute critical actions that influence outcomes, such as evading defenders during attacks, exchanging passes with teammates, and adjusting shots towards the goal [1,2]. These actions occur under time constraints and highly competitive conditions [3]. Therefore, the ability to rapidly assess situations and execute changes in direction and speed is crucial for athletic performance. These characteristics are closely associated with agility [4]. Agility is defined as ‘rapid whole-body movement with change of velocity or direction in response to a stimulus’, and its components include change-of-direction speed (CODS) and perceptual and decision-making factors [4]. CODS primarily depends on lower-limb muscle strength [5], whereas perceptual and decision-making factors are related to cognitive function [6]. Therefore, improving lower-limb muscle and cognitive function may enhance agility.

Soccer is characterised by an intermittent exercise pattern in which short bursts of high-intensity exercise alternate with periods of low-intensity exercise [7]. Players frequently perform repeated high-intensity sprints during matches [8]. However, high-intensity exercise temporarily impairs executive functions, such as attentional control, logical reasoning, and decision making [9]. Error rates in the Stroop task increase with exercise intensity [10]. Such cognitive decline may involve reduced neural activity due to decreased oxygenation in the prefrontal cortex [11,12]. However, changes in cognitive function cannot be explained solely by variations in ambient oxygen concentration. Recent evidence suggests that exercise-induced changes in cognitive function, particularly those related to executive function and reactive processes, are more closely related to neurovascular mechanisms than to ambient oxygen concentration [13]. Furthermore, high-intensity exercise negatively affects cognitive and muscle function. Muscle fatigue associated with repeated contractions may contribute to this decline, partly through reduced Ca^2+^ release from the sarcoplasmic reticulum (SR) [14]. Furthermore, high-intensity exercise may reduce arterial blood oxygen concentration, potentially limiting oxygen delivery to the muscles [15]. Consequently, these effects could reduce agility by impairing cognitive and lower-limb muscle function.

Recently, there has been increased interest in natural dietary supplements for improving athletic performance and recovery [16]. Beetroot juice (BJ) is a naturally derived food rich in nitrate (NO_3_^−^). Ingested NO_3_^−^ is reduced to nitrite (NO_2_^−^) by oral anaerobic bacteria and subsequently exerts physiological effects via conversion to nitric oxide (NO) [17,18]. NO has vasodilatory effects and can increase blood flow and oxygen delivery to the brain and skeletal muscles, thereby enhancing neurological and muscular function [19,20,21,22]. Additionally, NO increases local cerebral blood flow, enhances prefrontal cortex oxygenation, and may attenuate cognitive performance decline following high-intensity exercise [22,23]. Furthermore, NO may improve muscle strength and power output by promoting Ca^2+^ release from the SR and increasing microvascular PO_2_ (PO_2_mV) [21,24,25,26]. These effects may be more pronounced in fatigued states [27].

To our knowledge, only one study has directly examined BJ supplementation and reactive agility performance [28]. Rogers et al. reported improvements in response time during reactive agility drills, whereas simple reaction time assessed at rest was unchanged; the authors suggested that this may reflect already optimal endothelial function and preserved blood flow at rest in young, healthy adults. In contrast, one study examined the effects of BJ supplementation on change-of-direction performance, primarily interpreting outcomes through neuromuscular mechanisms rather than the perceptual–cognitive components of reactive agility [29]. Agility is determined not only by the muscle function supporting directional changes but also by cognitive functions such as perception of external stimuli and decision making [5,6]. During matches, soccer players frequently perform abrupt changes in direction, such as evading opponents, in response to movements following high-intensity linear sprints [30,31]. Such high-intensity sprints may impair agility by temporarily reducing cognitive and muscular functions owing to localised oxygen deficiency [11,12,14,15]. BJ may improve agility by enhancing cognitive function and lower-limb muscle function. Furthermore, the reduction of NO_2_^−^ to NO is enhanced under conditions of local hypoxia [32,33]. Therefore, the effects of BJ supplementation may be most apparent during reduced oxygenation that occurs immediately after high-intensity sprinting. Accordingly, we incorporated an all-out sprint protocol and assessed agility ability immediately post-sprint using soccer-specific agility and change-of-direction tests.

This study aimed to examine the effects of acute BJ intake on agility and change-of-direction performance immediately after high-intensity sprinting. We hypothesised that acute BJ intake suppresses the decline in agility and change-of-direction performance following high-intensity sprinting.

## 2. Materials and Methods

### 2.1. Study Design

**Figure 1:**
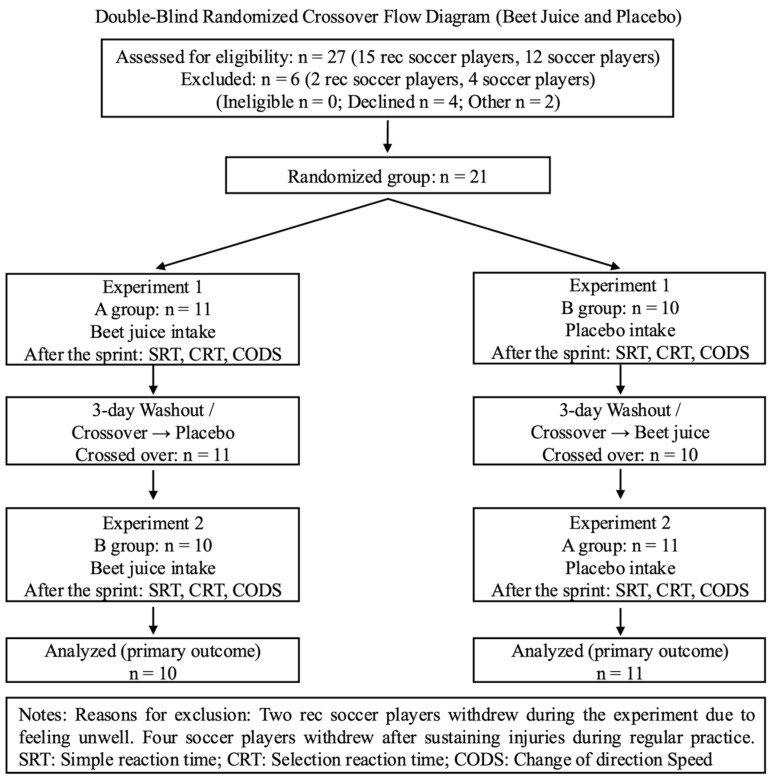
CONSORT flow diagram for a double-blind randomized crossover design. Twenty-one individuals were assessed for eligibility; six were excluded (four people were injured during routine practice; two people felt unwell during the experiment). Twenty-one participants were randomized (A group: BJ → PL, *n* = 11; B group: PL → BJ, *n* = 10), completed both periods, and were included in the primary analysis.

This study used a double-blind, randomised, crossover design (Figure 1). Participants were randomly assigned to Group A or Group B. Group A consumed BJ in the first experiment and a placebo (PL) in the second. Conversely, Group B consumed PL in the first experiment and BJ in the second. For each experiment, both beverages were processed to be indistinguishable in appearance and transferred to identical containers to ensure visual similarity. Furthermore, a third party (a graduate student from the same department), who was not involved in data analysis, implemented the double-blind procedure by labelling the beverages. Information regarding supplement assignment and distribution remained confidential until all trials were completed. Previous studies have reported that the effects of nitrate intake dissipate within 72 h [28]. A 3-day washout period was implemented to minimise residual effects. Written informed consent was obtained from all participants prior to data collection. All experimental procedures complied with the Declaration of Helsinki and were approved by the Kyushu University Graduate School of Human and Environmental Studies, Department of Health and Sports Science Ethics Committee (Project No. HSS-202502, date: 23 July 2025). For studies involving humans, and registered in the University Hospital Medical Information Network (ID: UMIN000060485; date: 27 January 2026; accessed on 27 January 2026). The primary analysis focuses on differences in simple response time (SRT), choice response time (CRT), and change-of-direction speed (CODS) following BJ or PL intake. This study was conducted as an experimental research study in the field of exercise and physiology and is not intended for medical purposes.

**Figure 2:**
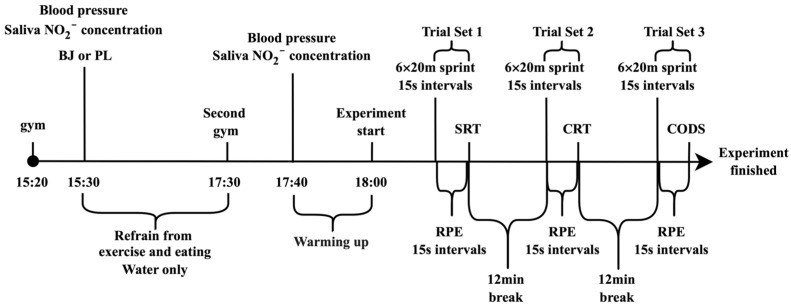
Schematic Diagram of the Experimental Protocol. BJ: beetroot juice, PL: placebo, SRT: simple response time, CRT: choice response time, CODS: change-of-direction speed.

The protocol for the day of the experiment is shown below (Figure 2): On the day of the experiment, participants refrained from exercise and gathered at the Kyushu University General Gymnasium at 15:20. After arriving, participants rested for 10 min before blood pressure and salivary NO_2_^−^ concentration were measured. Subsequently, participants consumed either BJ or PL. Since it has been reported that blood concentrations peak approximately 2 h after NO_3_^−^ intake [34], participants were allowed free activity for 2 h post-ingestion. During this period, exercise was prohibited, and participants were permitted to consume only water. After 2 h, participants reassembled. Following another 10-min rest period, blood pressure and salivary NO_2_^−^ concentration were measured again. Participants then completed a 20-min warm-up. Subjective exercise intensity, assessed using the rating of perceived exertion (RPE), was recorded after the warm-up, and the experiment then commenced. The experiment comprised three sets and was conducted on an artificial turf field adjacent to the Kyushu University General Gymnasium (Air temperature: 29.93 ± 2.01 °C; humidity: 72 ± 10.54%; Japan Meteorological Agency). All participants wore soccer cleats. In the first set, participants performed 6 × 20-m maximal sprints at 15-s intervals [35], and yoga was permitted after each sprint. RPE and SRT were measured immediately after each sprint (within 15 s). After a 12-min rest following the first set [36], the second set repeated the same six sprints, followed by measurements of RPE and CRT. In the third set, RPE and CODS were measured after sprinting. In each experimental session, participants performed 18 sprints and completed three agility tests. The initial session aimed to familiarise participants with each test component. Participants performed each test only once after the warm-up to minimise learning effects. During the experiment, participants consumed only water for hydration.

### 2.2. Participants

Initially, 12 male soccer players were recruited from Kyushu University soccer clubs. However, four participants withdrew owing to injuries sustained during training. Therefore, 13 additional male recreational soccer players were recruited, resulting in 21 outfield participants (Table 1). An a priori power analysis was conducted using G*Power (Version 3.1.9.6) to determine the required sample size. According to Rogers et al. [28], the effect sizes of BJ on agility were *d* = 0.92 for the semi-circle drill and *d* = 0.97 for the Get-up-and-go drill. Therefore, the effect size for BJ in this study was set at *d* = 0.90. Sample size estimation was based on a paired *t*-test, with *d* = 0.9, *α* = 0.05, and 1 − *β* = 0.8. This analysis indicated that a minimum sample size of 12 participants was required to achieve adequate statistical power. All participants were non-smokers, were not taking any regular medication, had no history of orthopaedic injury within the past 6 months, and were not currently taking supplements containing BJ or NO. Participants were instructed to abstain from alcohol, caffeine, and nicotine for at least 6 h before the experiment [28]. Additionally, participants were asked to avoid oral disinfectants or mouthwashes for 24 h before the experiment [37]. From two days before the experiment until the test day [38], and throughout the washout period, participants were requested to avoid consuming foods with a high nitrate content. All experiments were conducted under standardised conditions within the same time window (±1 h).

### 2.3. Test Substances

In the BJ intake experiment, participants consumed a single 70 mL dose of concentrated beetroot juice (BEET IT Sport Pro-Elite Shot Nitrate 400, James White Drinks, Ipswich, UK) [28]. BJ contained approximately 400 mg of NO_3_^−^. In the placebo intake experiment, participants consumed an equal volume of vegetable juice with a similar flavour (Vegetable Juice 100 Original, Kagome, Nagoya, Japan). The PL beverage contained NO_3_^−^ at 11.38 ± 17.72 mg/100 g [39]. The PL beverage also contained naturally derived carotenoids. Although carotene has antioxidant effects, continuous long-term intake is necessary for these effects to manifest [40]. The nutritional components are listed in Table 2. Since there was an 8.8 g difference in carbohydrate content between PL and BJ that could potentially affect the experimental results, 10 g of dextrin (maltodextrin, Nichiga, Takasaki, Japan) was added to PL.

### 2.4. Salivary Nitrite Concentration

Salivary NO_2_^−^ concentration was qualitatively assessed using salivary test strips (Nitric Oxide Indicator Strips, Jilin Hongyi Bio-Tech Co., Ltd., CHN, Changchun, China). Specifically, the test strip was placed on the tongue surface to absorb saliva and held for 10–15 s. The result was determined based on the colour change in the test strip. NO levels were qualitatively assessed by comparing the test strip colour with the six-step colour scale printed on the bottle: “1: depleted (10 μmol/L); 2: low (20 μmol/L); 3: threshold (110 μmol/L); 4: target (220 μmol/L); 5: high (435 μmol/L); 6: very high (870 μmol/L)”. Measurements were taken under the following two conditions to confirm the effects of BJ intake: 1: pre-ingestion assessment to confirm that all participants started with similarly low salivary NO_2_^−^ levels; 2: approximately 2 h post-BJ intake to record changes in NO_2_^−^ availability following supplementation.

### 2.5. Blood Pressure

BJ intake has been reported to lower blood pressure [34]. Therefore, measurements were performed using an automatic blood pressure monitor (HEM-1021, Omron Corporation, JPN, Kyoto, Japan) to confirm the effect of BJ intake and avoid excessive reductions in blood pressure. Participants remained seated at rest for 10 min before measurement to standardise conditions. Supplementation was evaluated approximately 2 h after BJ intake, and systolic blood pressure (SBP) and diastolic blood pressure (DBP) readings were recorded.

### 2.6. Sprint Running Intensity and Duration

The Borg scale [41], a subjective exercise intensity indicator (6–20), was used to evaluate sprint running intensity. The assessments were conducted at the end of the warm-up period and immediately after each sprint. Additionally, the duration of each sprint was recorded to confirm that participants performed the sprints appropriately.

### 2.7. Agility and Change-of-Direction Drill Measurement

**Figure 3:**
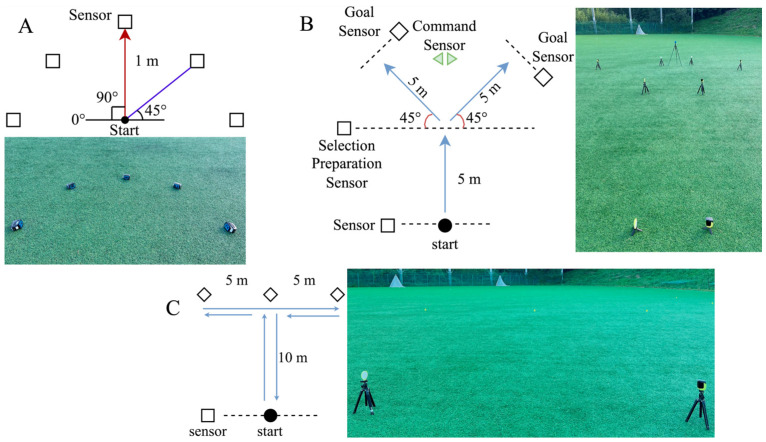
Schematic Diagram of Agility and Change-of-Direction Tests. (**A**): semi-circle drill; (**B**): Y-Shaped agility test; (**C**): T-Test.

Agility and change-of-direction tests were conducted using an agility measurement system (WITTY-SEM; Microgate Srl, Bolzano, Italy). Since soccer players exhibit significantly more foot movements than hand movements, the semi-circle drill developed by Čoh et al. [42] was used for SRT measurement. Participants reacted to the visual stimuli by quickly moving their feet in front of the sensors, and reaction time was measured (Figure 3A). This measurement method utilises five sensors arranged in a semi-circle on the floor. Each sensor was positioned 1 m from the centre at the following angles: two at 0°, two at 45°, and one at 90°. Upon initiation of the drill, the sensors were lit 15 times in random order. Participants were instructed to move to the lit sensor as quickly as possible, step once on the ground in front of the sensor with one foot, and return to the centre before the next sensor was lit. Reaction times were measured for all 15 trials, and the mean was calculated.

The CRT was measured using the Y-shaped agility test (Figure 3B), as described by Oliver and Meyers [43]. This test aims to evaluate cognitive processing abilities during movement and has been reported to be highly reliable in soccer players. Specifically, the starting point was defined using a choice-readiness sensor placed 5 m ahead. Additionally, goal sensors were placed 5 m forward, at 45°to the left and right of the choice-readiness sensor. Direction sensors were positioned between the choice-readiness sensor and each goal sensor. After passing the choice-readiness sensor, participants were required to pass through the goal sensor indicated by the direction sensor as quickly as possible. This test was conducted in triplicate. The completion time for each test was recorded, and the mean value was calculated. In this study, SRT and CRT represent total response time, including movement execution rather than pure reaction time.

During matches, soccer players must perform not only forward sprints but also diverse movements, such as side-steps and back-steps. Therefore, the T-test was used to evaluate the CODS [44]. The procedure for this test is illustrated in Figure 3C. First, sensors were placed at the starting point. The central marker was positioned 10 m away from the starting point. The left and right markers were then placed 5 m to the left and right of the central marker. The participants sprinted from the starting point to the central marker. Next, the participant moved to the left marker using side-steps, followed by movement to the right marker using side-steps (or vice versa). Subsequently, the participant returned to the centre marker and then to the starting point. When moving to each marker, participants were required to touch the marker with their hands. The course layout and test procedure were explained to the participants before the experiment. The test was performed twice, completion times were recorded, and the mean value was calculated.

### 2.8. Statistical Analysis

All statistical analyses were performed using SPSS version 27.0.0.0 (IBM, Armonk, NY, USA). As the data were not normally distributed, as determined by the Shapiro–Wilk test, changes in sprint time and RPE over time were assessed using the Friedman test. When significant differences were identified, multiple comparisons were performed using Dunn’s procedure with Bonferroni adjustment. Changes in salivary NO_2_^−^ concentration and blood pressure, as well as completion times for the SRT, CRT, and CODS tests, were analysed using the Wilcoxon signed-rank test. To evaluate the effect size of BJ, the effect size r for the Wilcoxon signed-rank test was calculated using the following equation:

$$r=\frac{n}{\sqrt{Z}}$$

where *Z* is the *Z* value from the Wilcoxon test and *n* is the sample size. An *r* value of 0.1 was interpreted as a small effect, 0.3 as a moderate effect, and 0.5 as a large effect [45].

In response to the use of non-parametric tests due to non-normality, an a priori power analysis was repeated using G*Power (version 3.1.9.6) for a two-tailed Wilcoxon signed-rank test (*d* = 0.9, *α* = 0.05, and 1 − *β* = 0.8). This provided a required sample size of 13 participants; therefore, the present sample size (*n* = 21) was considered adequate. Subgroup analyses by competitive level were conducted for exploratory purposes only and were not included in the a priori power calculation. Data were presented as mean ± standard deviation, and the significance level was set at *p* < 0.05.

## 3. Results

### 3.1. Salivary NO_2_^−^ Concentration

Following the BJ intake, NO_2_^−^ concentration increased significantly in all participants (*p* < 0.001; *r* = 0.63). In contrast, no significant change in NO_2_^−^ concentration was observed after PL intake (*p* = 0.782; *r* = 0.04) (Table 3).

### 3.2. Changes in Blood Pressure

A significant decrease in SBP was observed in all participants after BJ administration (*p* < 0.001; *r* = 0.80). In contrast, no significant change was observed in DBP (*p* = 0.241; *r* = 0.26). Furthermore, after PL intake, no significant change was observed in SBP (*p* = 0.411; *r* = 0.18) or DBP (*p* = 0.134; *r* = 0.33) (Table 4).

### 3.3. Sprint Time Between Sets and RPE

No significant differences in sprint time between sets were observed for any participant in either the BJ group (*p* = 0.109) or the PL group (*p* = 0.637). Significant differences in the RPE were observed between the BJ and PL groups (BJ: *p* < 0.001; PL: *p* < 0.001). Post hoc tests revealed that Trial Set 1 values were significantly lower than those of Trial Set 3 (BJ: *p* = 0.005; PL: *p* = 0.008) (Table 5).

### 3.4. SRT, CRT, CODS

**Figure 4:**
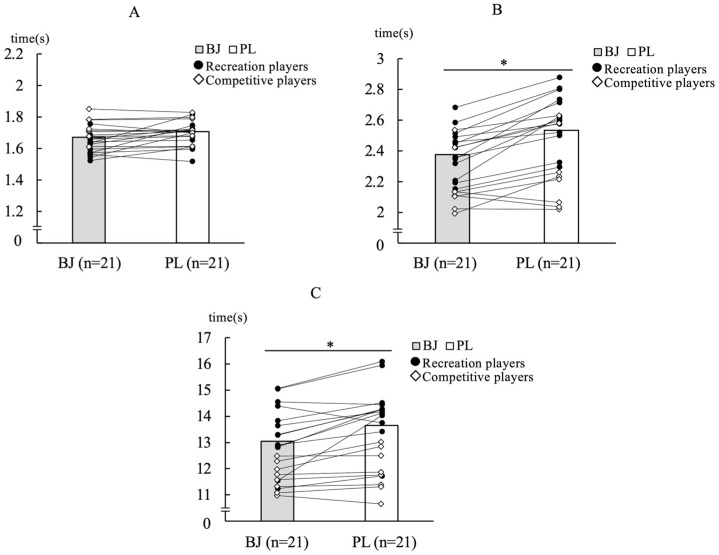
The figure shows the agility test results after a sprint between beetroot juice intake and placebo intake. Grey is BJ (beetroot juice), White is PL (placebo), The filled circles represent recreational players, whereas the open diamonds denote competitive players, (**A**): Completion times of the semi-circle drill; (**B**): Completion times of the Y-Shaped agility test; (**C**): Completion times of the T-Test; * *p* < 0.05, effect sizes (*r*): small (*r* = 0.10), medium (*r* = 0.30), large (*r* = 0.50).

No significant difference was observed in the SRT after BJ intake (*p* = 0.185; *r* = 0.05) (Figure 4A). Analysis of the separated data showed significant difference between recreational soccer players (*n* = 13) after BJ and PL intake (*p* = 0.028; *r* = 0.13). CRT was significantly faster after BJ intake than after PL intake in the combined data (*p* < 0.001; *r* = 0.74) and recreational soccer players (*p* < 0.001; *r* = 0.84) (Figure 4B). In competitive players (*n* = 8), a moderate effect size was observed (*p* = 0.016; *r* = 0.49). However, this exploratory subgroup finding should be interpreted with caution due to the small sample size. CODS was significantly faster after BJ intake than after PL intake (*p* < 0.001; *r* = 0.47) (Figure 4C). Analysis of the separated data showed that CODS was significantly faster after BJ intake than after PL intake among recreational soccer players (*p* < 0.001; *r* = 0.52).

### 3.5. Verification of Subject Identification

Participants completed a free-response questionnaire about taste and product characteristics after BJ or PL intake to verify whether the observed effect was due to psychological factors or physiological effects of BJ. Among all soccer players (*n* = 21) in the study, the combined intake in the BJ group mainly included tomato carrot juice (5 people), tomato juice (4 people), and Yasai Seikatsu + sugar (3 people), while smaller numbers consumed carrot juice, plain Yasai Seikatsu, carrot lemon juice, orange carrot juice, spinach carrot juice, pumpkin caramel juice, tamarind juice, plum juice, and berry-based juice (1 person each). In the PL group, the most frequent choice was Yasai Seikatsu (6 people), followed by general vegetable juice (5 people), tomato carrot juice (3 people), orange carrot juice (3 people), carrot juice (2 people), tomato juice (1 person), and carrot pineapple juice (1 person). None of the participants who consumed BJ identified it accurately. Conversely, 29% of participants who consumed PL identified it accurately.

## 4. Discussion

The primary findings of this study indicate that BJ intake improved post-sprint performance in terms of CRT and CODS, whereas SRT did not differ significantly between BJ and PL intake. These results provide new evidence that BJ may improve agility-related performance in sports requiring rapid decision making and change-of-direction movements, particularly under post-sprint conditions.

According to Lefferts et al. [38], a significant colour change was observed in saliva test strips 2 h after BJ intake. In this study, salivary NO_2_^−^ concentration also significantly increased 2 h after BJ intake. Furthermore, Wylie et al. [34] confirmed that blood NO_3_^−^ concentration peaks at least 2 h after BJ consumption, suggesting the intake protocol used in this study may maximise NO_3_^−^ bioavailability. NO_3_^−^ is taken up and concentrated by the salivary gland membrane protein sialin [46]. Concentrated NO_3_^−^ is secreted from the salivary glands into saliva, where it is reduced to NO_2_^−^ by anaerobic bacteria on the posterior tongue [47]. This mechanism enables the detection of increased salivary NO_2_^−^ concentrations using saliva testing. In addition, prior studies have reported that BJ consumption lowers SBP and DBP [34]. In this study, SBP decreased by approximately 10 mmHg after BJ consumption. NO generated via the NO_3_^−^–NO_2_^−^–NO pathway promotes cyclic guanosine monophosphate-mediated vasodilation, reducing peripheral vascular resistance [48,49]. However, the absence of a DBP reduction did not align with our initial hypothesis. One possible reason is that the participants’ baseline DBP was approximately 65 mmHg, which is close to the lower limit of the normal range (60 mmHg), leaving little room for further reduction. Excessive NO is rapidly oxidised and excreted renally as NO_3_^−^ via these pathways [18], potentially limiting further reductions in DBP. Although the salivary test strips provide only a qualitative estimate rather than a laboratory-based concentration, the concurrent reduction in SBP demonstrates that the intervention achieved its intended physiological effect and that NO_3_^−^ bioavailability was maintained.

No significant differences were observed in sprint performance across the three sets. Consistent with Reynolds et al. [50], acute NO_3_^−^-rich BJ ingestion did not improve short-duration repeated-sprint running performance despite clear elevations in circulating NO_3_^−^, suggesting limited ergogenic benefit for very brief, near-maximal sprint tasks. In contrast, BJ may be more beneficial for more complex, agility-related actions that require greater perceptual–cognitive processing. This pattern is consistent with that of Rogers et al. [28], who reported improvements in reactive agility drills but no change in resting simple reaction time. Significant differences in RPE were observed between sets; even under identical exercise loads and adequate rest conditions, the third set showed a higher RPE than did the first set. This finding is consistent with findings that RPE increases over time, even under the same load [51]. Factors contributing to increased RPE may include baseline fitness levels and psychological factors associated with the final set (e.g., perceived fatigue and awareness of all-out effort). Therefore, both the sprint time and RPE indicated that the participants performed the sprint runs in accordance with the protocol.

Immediately after all-out sprinting, SRT performance did not differ between BJ and PL in all included participants. Exploratory subgroup patterns suggested a modest improvement in recreational players; however, these subgroup observations are hypothesis-generating and should be interpreted cautiously, given the limited sample size, particularly in the competitive players. Rogers et al. [28] reported improved performance in a semi-circle drill, comparable to our SRT task, in physically active healthy men under non-fatiguing conditions; they discussed neuromuscular contributions as a potential mechanism. In the present study, SRT was assessed immediately post-sprint, when performance may depend on perceptual–cognitive and coordinative demands in addition to muscle function. Although we did not directly assess cognitive function or cerebral oxygenation, prior studies indicate that high-intensity exercise can transiently alter prefrontal oxygenation and impair cognitive performance [11,12]. Because NO_3_^−^-derived NO production is enhanced under hypoxic conditions [32], one plausible explanation is that BJ may support cerebral perfusion/oxygenation and help maintain post-sprint perceptual–cognitive function. Consistent with this possibility, NO_3_^−^ supplementation has been associated with increased prefrontal tissue oxygenation during cycling time trials [23] and with the attenuation of post-exercise cognitive decline during high-intensity intermittent exercise [52]. The smaller apparent response in competitive players may relate to differences in training status and the relative internal load imposed by the sprint protocol. Although RPE increased markedly after the all-out sprints in all participants, competitive players likely have greater tolerance to repeated sprint demands due to long-term training adaptations, which may result in a lower relative internal load at the same external workload. Consequently, post-sprint disruption to perceptual–cognitive performance may be smaller in competitive players [13], potentially limiting the scope for BJ-related benefit. Future studies with larger competitive cohorts and direct measures of internal load (e.g., heart rate, lactate), as well as cognitive function and cerebral oxygenation, are needed to test these hypotheses.

CRT reflects choice-based processing and was assessed using the Y-test in the present study. Importantly, the Y-test completion time represents a composite of multiple components, including sprinting from the start, responding to the stimulus, and crossing the finish line; therefore, performance is unlikely to be explained by a single factor. To the best of our knowledge, no previous studies have evaluated Y-test performance following BJ intake; therefore, the present findings extend the literature by considering both perceptual–cognitive and neuro-muscular contributions. As discussed above, NO_3_^−^-derived NO production may be enhanced under reduced oxygenation [32], suggesting that BJ could help maintain prefrontal tissue oxygenation and preserve stimulus processing or decision making during post-sprint testing [23]. From a neuromuscular perspective, NO_3_^−^ supplementation is associated with increased muscle blood flow and vascular conductance, particularly in type II fibres [20], and may increase microvascular PO_2_ in these fibres [21], which could support repeated sprint actions within the Y-test and contribute to faster overall completion times. These synergistic effects significantly improved Y-test performance in recreational soccer players (*r* = 0.84). In competitive players (*n* = 8), a moderate effect size was observed (*r* = 0.49); however, this exploratory subgroup finding should be interpreted with caution. In the extant literature on motor learning, reaction time (from stimulus onset to movement initiation) and response time (from stimulus onset to task completion) are often distinguished. The agility-based assessment method employed in this study reflects a response time measurement that integrates the processes of perception, decision making, and movement execution.

Unlike SRT and CRT, CODS does not require stimulus-driven response; rather, it involves executing predetermined movement patterns and is often used as an indicator of pre-planned change-of-direction performance with a strong neuromuscular contribution [5]. In the present study, CODS improved following BJ intake in all participants, suggesting a potential benefit for change-of-direction performance under post-sprint conditions. Notably, findings in the literature are not uniform; for example, López-Samanes et al. [29] reported no clear improvement in CODS after BJ intake. One possible explanation is that their protocol did not include an immediate high-intensity sprint stimulus and may not have produced the transient, localised reductions in muscle oxygenation under which NO_3_^−^-derived NO production is enhanced [32]. Consistent with this interpretation, BJ may support change-of-direction performance by enhancing muscle function, particularly in type II fibres. Haider and Folland [25] reported greater force production during the early phase of contraction following BJ intake, and Wylie et al. [26] observed increased power output during repeated high-intensity efforts. Mechanistically, NO_3_^−^ supplementation has been linked to improved excitation–contraction coupling in type II fibres, including greater Ca^2+^ handling capacity [24], and increased PO_2_mV in type II fibres during exercise [21]. Because repeated all-out sprints can induce a transient, localised hypoxic environment within active muscle, NO_3_^−^-derived NO production may be enhanced under these conditions, potentially supporting perfusion and contractile function. Exploratory subgroup patterns suggested that the CODS improvement appeared more evident among recreational players, whereas competitive players showed smaller changes descriptively; however, these subgroup observations should be interpreted cautiously, given the limited competitive sample size. Future studies with larger competitive cohorts and direct measures of neuromuscular and physiological responses are warranted to clarify whether competitive players exhibit smaller effects or whether the present subgroup patterns reflect limited sample size.

Taste and product questionnaires were administered after each intake to verify the physiological effects of BJ and minimise psychological effects. The results showed that none of the participants accurately identified BJ, confirming that they had no prior experience with BJ. Conversely, 29% of the participants who consumed PL accurately identified PL. Although BJ was not identified, participants who recognised PL may have been able to distinguish BJ from PL. However, as most participants merely recognised it as ‘juice’, the influence was considered limited, and the likelihood that psychological factors affected the performance results was deemed small. It is difficult to distinguish whether the effects observed in the 29% of participants who identified PL were due to the physiological effects of BJ or to psychological effects related to participant identification.

This study has some limitations. The exercise intensity imposed in this study may have been relatively low for soccer players. When the study was conducted outdoors with many participants, it was difficult to adjust the exercise intensity according to each participant’s fitness level. Future studies should determine exercise loads based on individual fitness levels. The experiment was conducted outdoors in hot environments. Therefore, the effects of BJ intake during winters or in cold conditions could not be examined in this study. Although 12-min rest periods between sets were implemented in this study, which may have helped to maintain performance, it is difficult to confirm the elimination of fatigue effects. Therefore, it cannot be ruled out that altering the order of the agility test administration could yield different results. Consequently, future studies should develop protocols that more rigorously control fatigue and order effects. The agility measurement device used in this study, which represented the first attempt to evaluate the semi-circle drill, had difficulty detecting black shoes. The device failed to register instances where participants stepped quickly in front of the sensor. Future studies should require participants to wear bright-coloured shoes. Participants affected by this technical issue were retested 1 week later to minimise potential learning effects, and the retest data were used for the final analyses. Challenges remain regarding methods for evaluating sprint intensity. This study used sprint speed and RPE; however, sprint speed may lack objectivity because participants could self-regulate the speed. Similarly, RPE is a subjective indicator, which limits its reliability. In addition, salivary NO_2_^−^ was assessed using colourimetric test strips, which provide only a qualitative estimate rather than laboratory-based concentrations. Future studies should incorporate objective biochemical assays alongside objective intensity measures. Initially, heart rate monitors were planned but were not used because the duration of the six sprints was too short to allow accurate heart rate measurement. Future studies should consider incorporating objective measures (e.g., heart rate and blood lactate concentration). The competitive subgroup was small (*n* = 8), limiting subgroup inference; future studies should include larger competitive samples. Finally, the ability to distinguish between BJ and PL is the most critical issue. Matching the taste of BJ and PL is essential to accurately assessing the physiological effects of BJ. Future research requires improved experimental designs to minimise psychological bias arising from participants’ identification.

## 5. Conclusions

BJ intake suppressed performance decline in all tests immediately after high-intensity sprinting in recreational soccer players. In contrast, only CRT improved significantly in the soccer players. From a practical perspective, BJ has the potential to be an effective performance-enhancing tool for recreational soccer players and athletes, particularly in sports where agility is critical (e.g., basketball). BJ is a concentrated beverage made from natural vegetables, a safe source of inorganic NO_3_^−^; its use is supported by established scientific evidence regarding its role in enhancing athletic performance. Furthermore, BJ is commercially available and readily accessible to consumers. Therefore, BJ appears promising for enhancing agility-related athletic performance. However, to confirm these findings and establish broader applicability, further research with larger sample sizes and practice-oriented experimental designs is necessary.

## Figures and Tables

**Table 1:** Subject Characteristics. The left side shows all participants. The right side shows participants grouped by competitive level.

	Participants (*n* = 21)	Recreation (*n* = 13)	Soccer Player (*n* = 8)
Age (years)	23.7 ± 3.6	26.1 ± 2.4	19.8 ± 1.0
Height (cm)	174.3 ± 6.3	177.5 ± 4.5	169.1 ± 5.4
Weight (kg)	69.9 ± 10.4	75.5 ± 9.1	60.9 ± 5.2
BMI (kg/m^2^)	22.9 ± 2.6	24.0 ± 2.8	21.3 ± 1.4
Athletic experience (years)	6.5 ± 4.8	3.1 ± 1.6	12.0 ± 2.7
Weekly training sessions (times)	3.4 ± 1.9	1.7 ± 0.8	5.4 ± 0.5

**Table 2:** Nutrition Facts Label.

Amount per 100 mL	BJ	PL
Energy	83 kcal	33 + 36.9 kcal
Protein	2.9 g	0.4 g
Fat	0.1 g	0 g
Carbohydrates	17 g	8.2 + 9.4 g
Sugars (Total)	N/A	7.7 g
Sugars (Free)	13 g	6.5 g
Dietary Fiber	N/A	0.1~0.8 g
Sodium (as Salt Equivalent)	0.41 g	0~0.2 g
Potassium	N/A	99~260 mg
Calcium	N/A	6~15 mg
Vitamin A	N/A	220~630 μg
Vitamin C	N/A	25~94 mg
Vitamin K	N/A	0~6 μg
α-Carotene	N/A	710~3100 μg
β-Carotene	N/A	2300~5900 μg
Nitrate	400 mg	11.38 ± 17.72 mg

N/A: Unknown.

**Table 3:** Changes in Salivary NO_2_^−^ Concentration before and after BJ and PL intake in all participants.

Group	*n*	Pre	Post	*Z*	*p*-Value	*r*
BJ	21	1.9 ± 0.8	5.7 ± 0.5	−4.064	0.000 *	0.63
PL	21	1.9 ± 0.6	1.9 ± 0.7	−0.277	0.782	0.04

BJ: beetroot juice; PL: placebo, * *p* < 0.05, effect sizes (*r*): small (*r* = 0.10), medium (*r* = 0.30), large (*r* = 0.50).

**Table 4:** Changes in blood pressure before and after BJ and PL intake in all participants.

Group (mmHg)	*n*	Pre	Post	*Z*	*p*-Value	*r*
BJ SBP	21	120.1 ± 10.9	110.3 ± 7.1	−3.686	0.000 *	0.80
PL SBP	21	118.1 ± 8.6	119.3 ± 9.9	−1.172	0.241	0.26
BJ DBP	21	65.5 ± 7.7	64.3 ± 6.7	−0.823	0.411	0.18
PL DBP	21	64.8 ± 8.5	67.1 ± 8.6	−1.497	0.134	0.33

BJ SBP: Systolic blood pressure after beetroot juice intake; PL SBP: Systolic blood pressure after placebo intake; BJ DBP: Diastolic blood pressure after beetroot juice intake; PL DBP: Diastolic blood pressure after placebo intake, * *p* < 0.05, effect sizes (*r*): small (*r* = 0.10), medium (*r* = 0.30), large (*r* = 0.50).

**Table 5:** Sprint Time and RPE for the three trial sets.

	Trial Set	1	2	3	*χ* ^2^	*p*-Value
Sprint Time	BJ(s)	3.77 ± 0.43	3.76 ± 0.44	3.72 ± 0.46	4.424	0.109
	Mean Rank	2.08	2.07	1.85		
	PL(s)	3.79 ± 0.41	3.80 ± 0.45	3.79± 0.49	0.903	0.637
	Mean Rank	1.93	2.02	2.04		
RPE	BJ	16.1 ± 2.4	17.5 ± 2.0	18.0 ± 1.6	53.784	0.000
	Mean Rank	2.24	3.19	3.57		
	PL	16.6 ± 2.0	17.8 ± 2.0	18.4 ± 1.8	52.933	0.000
	Mean Rank	2.26	3.19	3.55		
Dunn–Bonferroni post hoc:		BJ Trial Set 1 < BJ Trial Set 3				0.005 *
		PL Trial Set 1 < PL Trial Set 3				0.008 *

BJ: beetroot juice; PL: placebo, * Dunn–Bonferroni post hoc tests *p* < 0.05.

## Data Availability

The original contributions presented in this study are included in the article. Further inquiries can be directed to the corresponding author.

## Abbreviations

The following abbreviations are used in this manuscript:

BJ	beetroot juice
PL	placebo
SRT	simple response time
CRT	choice response time
CODS	Change-of-direction speed
PO_2_mV	microvascular PO_2_
NO_3_^−^	nitrate
NO_2_^−^	nitrite
NO	nitric oxide
SBP	systolic blood pressure
DBP	diastolic blood pressure
SR	sarcoplasmic reticulum
RPE	Rating of Perceived Exertion

## References

[1] Henry G., Dawson B., Lay B., Young W. (2011). Validity of a reactive agility test for Australian football. Int. J. Sports Physiol. Perform..

[2] Roca A., Ford P.R., McRobert A.P., Williams A.M. (2013). Perceptual-cognitive skills and their interaction as a function of task constraints in soccer. J. Sport Exerc. Psychol..

[3] Čaprić I., Stanković M., Manić M., Preljević A., Špirtović O., Đorđević D., Spehnjak M., Damjan B., Sporis G., Trajkovic N. (2022). Effects of plyometric training on agility in male soccer players-a systematic review. J. Men’s Health.

[4] Sheppard J.M., Young W.B. (2006). Agility literature review: Classifications, training and testing. J. Sports Sci..

[5] Castillo-Rodríguez A., Fernández-García J.C., Chinchilla-Minguet J.L., Carnero E.Á. (2012). Relationship between muscular strength and sprints with changes of direction. J. Strength Cond. Res..

[6] Young W.B., Dawson B., Henry G.J. (2015). Agility and change-of-direction speed are independent skills: Implications for training for agility in invasion sports. Int. J. Sports Sci. Coach..

[7] Barnes C., Archer D.T., Hogg B., Bush M., Bradley P. (2014). The evolution of physical and technical performance parameters in the English Premier League. Int. J. Sports Med..

[8] Michailidis Y. (2022). The effectiveness of different training methods in soccer for repeated sprint ability: A brief review. Appl. Sci..

[9] Moore R.D., Romine M.W., O’Connor P.J., Tomporowski P.D. (2012). The influence of exercise-induced fatigue on cognitive function. J. Sports Sci..

[10] Labelle V., Bosquet L., Mekary S., Bherer L. (2013). Decline in executive control during acute bouts of exercise as a function of exercise intensity and fitness level. Brain Cogn..

[11] Chang H., Kim K., Jung Y.J., Kato M. (2017). Effects of acute high-intensity resistance exercise on cognitive function and oxygenation in prefrontal cortex. J. Exerc. Nutr. Biochem..

[12] Ochi G., Kuwamizu R., Suwabe K., Fukuie T., Hyodo K., Soya H. (2022). Cognitive fatigue due to exercise under normobaric hypoxia is related to hypoxemia during exercise. Sci. Rep..

[13] Nakao T., Hirata T., Adachi T., Fukuda J., Fukada T., Iino-Ohori K., Saito A. (2026). Cognitive Processing Efficiency (Throughput) Improves with Aerobic Exercise and Is Independent of the Environmental Oxygenation Level: A Randomized Crossover Trial. Sports.

[14] Karatzaferi C., Franks-Skiba K., Cooke R. (2008). Inhibition of shortening velocity of skinned skeletal muscle fibers in conditions that mimic fatigue. Am. J. Physiol. Regul. Integr. Comp. Physiol..

[15] Cairns S.P. (2013). Holistic approaches to understanding mechanisms of fatigue in high-intensity sport. Fatigue Biomed. Health Behav..

[16] Jiao X., Liu X., Cao Q., Deng Z. (2025). Plant-based supplements in enhancing exercise performance and recovery. Front. Nutr..

[17] Pannala A.S., Mani A.R., Spencer J.P., Skinner V., Bruckdorfer K.R., Moore K.P., Rice-Evans C.A. (2003). The effect of dietary nitrate on salivary, plasma, and urinary nitrate metabolism in humans. Free Radic. Biol. Med..

[18] DeMartino A.W., Kim-Shapiro D.B., Patel R.P., Gladwin M.T. (2019). Nitrite and nitrate chemical biology and signalling. Br. J. Pharmacol..

[19] Presley T.D., Morgan A.R., Bechtold E., Clodfelter W., Dove R.W., Jennings J.M., Kraft R.A., King S.B., Laurienti P.J., Rejeski W.J. (2011). Acute effect of a high nitrate diet on brain perfusion in older adults. Nitric Oxide.

[20] Ferguson S.K., Hirai D.M., Copp S.W., Holdsworth C.T., Allen J.D., Jones A.M., Musch T.I., Poole D.C. (2013). Impact of dietary nitrate supplementation via beetroot juice on exercising muscle vascular control in rats. J. Physiol..

[21] Ferguson S.K., Holdsworth C.T., Wright J.L., Fees A.J., Allen J.D., Jones A.M., Musch T.I., Poole D.C. (2015). Microvascular oxygen pressures in muscles comprised of different fiber types: Impact of dietary nitrate supplementation. Nitric Oxide.

[22] Wightman E.L., Haskell-Ramsay C.F., Thompson K.G., Blackwell J.R., Winyard P.G., Forster J., van der Giezen M., Kennedy D.O. (2015). Dietary nitrate modulates cerebral blood flow parameters and cognitive performance in humans: A double-blind, placebo-controlled, crossover investigation. Physiol. Behav..

[23] Fan J.L., Bourdillon N., Meyer P., Kayser B. (2018). Oral nitrate supplementation differentially modulates cerebral artery blood velocity and prefrontal tissue oxygenation during 15 km time-trial cycling in normoxia but not in hypoxia. Front. Physiol..

[24] Hernández A., Schiffer T.A., Ivarsson N., Cheng A.J., Bruton J.D., Lundberg J.O., Weitzberg E., Westerblad H. (2012). Dietary nitrate increases tetanic [Ca^2+^] i and contractile force in mouse fast-twitch muscle. J. Physiol..

[25] Haider G., Folland J.P. (2014). Nitrate supplementation enhances the contractile properties of human skeletal muscle. Med. Sci. Sports Exerc..

[26] Wylie L.J., Bailey S.J., Kelly J., Blackwell J.R., Vanhatalo A., Jones A.M. (2016). Influence of beetroot juice supplementation on intermittent exercise performance. Eur. J. Appl. Physiol..

[27] Tillin N., Moudy S., Nourse K., Tyler C. (2018). Nitrate supplement benefits contractile forces in fatigued but not unfatigued muscle. Med. Sci. Sports Exerc..

[28] Rogers R.R., Davis A.M., Rice A.E., Ballmann C.G. (2022). Effects of Acute Beetroot Juice Ingestion on Reactive Agility Performance. Oxygen.

[29] López-Samanes Á., Gómez Parra A., Moreno-Pérez V., Courel-Ibáñez J. (2020). Does acute beetroot juice supplementation improve neuromuscular performance and match activity in young basketball players? A randomized, placebo-controlled study. Nutrients.

[30] Caldbeck P., Dos’Santos T. (2022). A classification of specific movement skills and patterns during sprinting in English Premier League soccer. PLoS ONE.

[31] Daly L., Caulfield P., Martínez-Hernández D. (2025). Sprints, decelerations and turns most commonly precede goals in soccer: Analysis of 6 FIFA World Cups. Eur. J. Sport Sci..

[32] Gladwin M.T., Schechter A.N., Kim-Shapiro D.B., Patel R.P., Hogg N., Shiva S., Cannon R.O., Feelisch M., Lundberg J.O. (2005). The emerging biology of the nitrite anion. Nat. Chem. Biol..

[33] Van Faassen E.E., Bahrami S., Feelisch M., Hogg N., Kelm M., Kim-Shapiro D.B., Kozlov A.V., Kevil C.G., Patel R.P., Rifkind J.M. (2009). Nitrite as regulator of hypoxic signaling in mammalian physiology. Med. Res. Rev..

[34] Wylie L.J., Kelly J., Bailey S.J., Blackwell J.R., Skiba P.F., Winyard P.G., Jeukendrup A.E., Vanhatalo A., Jones A.M. (2013). Beetroot juice and exercise: Pharmacodynamic and dose-response relationships. J. Appl. Physiol..

[35] Gabbett T.J. (2010). The development of a test of repeated-sprint ability for elite women’s soccer players. J. Strength Cond. Res..

[36] Ishak A., Wong F.Y., Seurot A., Cocking S., Pullinger S.A. (2022). The influence of recovery period following a pre-load stimulus on physical performance measures in handball players. PLoS ONE.

[37] Govoni M., Jansson E.Å., Weitzberg E., Lundberg J.O. (2008). The increase in plasma nitrite after a dietary nitrate load is markedly attenuated by an antibacterial mouthwash. Nitric Oxide.

[38] Lefferts W.K., Hughes W.E., White C.N., Brutsaert T.D., Heffernan K.S. (2016). Effect of acute nitrate supplementation on neurovascular coupling and cognitive performance in hypoxia. Appl. Physiol. Nutr. Metab..

[39] Murata M., Ishinaga M. (2005). Nitrate and nitrite contents in vegetable juice, tea beverage and mineral water on the market. Shokuhin Eiseigaku Zasshi/J. Food Hyg. Soc. Jpn..

[40] Dadalt E.K., Stefani G.P. (2024). Effects of antioxidant vitamin supplementation on sports performance, endurance and strength performance: A systematic review and meta-analysis. Sport Sci. Health.

[41] Borg G.A. (1982). Psychophysical bases of perceived exertion. Med. Sci. Sports Exerc..

[42] Čoh M., Vodičar J., Žvan M., Šimenko J., Stodolka J., Rauter S., Maćkala K. (2018). Are change-of-direction speed and reactive agility independent skills even when using the same movement pattern?. J. Strength Cond. Res..

[43] Oliver J.L., Meyers R.W. (2009). Reliability and generality of measures of acceleration, planned agility, and reactive agility. Int. J. Sports Physiol. Perform..

[44] Pauole K., Madole K., Garhammer J., Lacourse M., Rozenek R. (2000). Reliability and validity of the T-test as a measure of agility, leg power, and leg speed in college-aged men and women. J. Strength Cond. Res..

[45] Cohen J. (1992). A power primer. Psychol. Bull..

[46] Qin L., Liu X., Sun Q., Fan Z., Xia D., Ding G., Ong H.L., Amarasinghe O., Foskett J.K., Wang S. (2012). Sialin (SLC17A5) functions as a nitrate transporter in the plasma membrane. Proc. Natl. Acad. Sci. USA.

[47] Hyde E.R., Andrade F., Vaksman Z., Parthasarathy K., Jiang H., Parthasarathy D.K., Ardissone A., Moffitt J.H., Petrosino J.F., Bryan N.S. (2014). Metagenomic analysis of nitrate-reducing bacteria in the oral cavity: Implications for nitric oxide homeostasis. PLoS ONE.

[48] Lgnarro L.J., Buga G.M., Wood K.S., Byrns R.E., Chaudhuri G. (1987). Endothelium-derived relaxing factor produced and released from artery and vein is nitric oxide. Proc. Natl. Acad. Sci. USA.

[49] Webb A.J., Patel N., Loukogeorgakis S., Okorie M., Aboud Z., Misra S., Rashid R., Miall P., Deanfield J., Benjamin N. (2008). Acute blood pressure lowering, vasoprotective, and antiplatelet properties of dietary nitrate via bioconversion to nitrite. Hypertension.

[50] Reynolds C.M.E., Evans M., Halpenny C., Hughes C., Jordan S., Quinn A., Hone M., Egan B. (2020). Acute ingestion of beetroot juice does not improve short-duration repeated sprint running performance in male team sport athletes. J. Sports Sci..

[51] Fusco A., Knutson C., King C., Mikat R.P., Porcari J.P., Cortis C., Foster C. (2020). Session RPE during prolonged exercise training. Int. J. Sports Physiol. Perform..

[52] Thompson C., Wylie L.J., Fulford J., Kelly J., Black M.I., McDonagh S.T., Jeukendrup A.E., Vanhatalo A., Jones A.M. (2015). Dietary nitrate improves sprint performance and cognitive function during prolonged intermittent exercise. Eur. J. Appl. Physiol..

